# Editorial: Metabolomics in human and animal ophthalmic research

**DOI:** 10.3389/fmolb.2025.1594647

**Published:** 2025-03-28

**Authors:** Diana Anna Dmuchowska, Muhaiyo Bartolo Khodjayeva, Michal Ciborowski

**Affiliations:** ^1^ Department of Ophthalmology, Medical University of Bialystok, Bialystok, Poland; ^2^ Mater Dei Hospital, Department of Ophthalmology, Medical University of Malta, L-Imsida, Malta; ^3^ Metabolomics and Proteomics Laboratory, Clinical Research Center, Medical University of Bialystok, Bialystok, Poland; ^4^ Department of Medical Biochemistry, Medical University of Bialystok, Bialystok, Poland

**Keywords:** metabolomics, aqueous humor (AH), LC-MS, NMR, GC-MS

Metabolomics offers valuable insights into the mechanisms of eye diseases, with the potential to improve diagnosis, treatment, and prevention. However, the role of small molecules in many ocular conditions remains unclear. This Research Topic includes studies performed on human samples to identify biomarkers and explore the promise and challenges of applying metabolomics in ophthalmology.

The extensive review by Belete et al. examines metabolomics research in eye disorders such as age-related macular degeneration, glaucoma, diabetic retinopathy, and myopia, highlighting its potential for biomarker discovery. As further discussed, recent metabolomics studies have revealed shared metabolic alterations across these major eye diseases. Disruptions in lipid metabolism, energy metabolism, arginine and proline metabolism pathways, and the perturbations in the urea cycle and oxidative stress suggest common mechanisms of disease progression and indicate potential therapeutic targets. These findings were drawn based on the analyses of plasma, serum, aqueous and vitreous humor, as well as tear fluid, using advanced analytical platforms widely used in metabolomics research, i.e., liquid and gas chromatography coupled to mass spectrometry (LC-MS and GC-MS, respectively) and nuclear magnetic resonance (NMR). However, challenges remain regarding standardization, cohort size, and the integration of multi-omics approaches.

The paper by Zemitis et al. investigates the metabolic alterations in pseudoexfoliation syndrome (PEX), which leads to various ocular complications, including glaucoma, lens subluxation, lens luxation, and increased risk of complications during cataract surgery. Through LC-MS metabolomic profiling of aqueous humor, the authors identify a distinct metabolic signature characteristic to PEX. The key findings include alterations in amino acid metabolism, energy production pathways, and oxidative stress markers, with specific disruptions in the urea cycle and mitochondrial function. The results indicate a potential link to ferroptosis, an iron-dependent programmed cell death. The authors suggest that ferroptosis may be implicated in the pathogenesis of PEX.

Another significant contribution by Kannan et al. integrates clinical and metabolomic analysis to identify molecular signatures, biomarkers, and therapeutic targets in primary angle-closure glaucoma (PACG). Utilizing aqueous humor and plasma samples, the research combined targeted metabolomic profiling (via LC-MS) with clinical assessments and cytokine analysis to identify metabolic remodelling involving immuno-metabolism, marked by elevated ATP, pro-inflammatory cytokines and distinct metabolites like fructose-6-phosphate and taurine. Significant overlap was observed between PACG metabolic pathways and those found in TNFα- and ATP-treated microglial cell models, reinforcing the role of neuroinflammation and microglial activation in PACG pathogenesis. These findings suggest ATP, cytokines, and other key metabolites as promising biomarkers, with P2 receptors, IDO1/2, and cytokine signalling emerging as potential therapeutic targets.

Schoumacher et al. present a 2-year longitudinal study on neovascular age-related macular degeneration (nAMD) patients. It is worth highlighting that the average number of visits per patient was almost eight, which is a rarity in metabolomics ophthalmic research. Plasma samples collected during the visits were analyzed using NMR metabolomics. The identified metabolomics biomarkers correlate with nAMD optical coherence tomography biomarkers (established routine diagnostic test). This study emphasizes the potential of metabolomics in monitoring disease progression and treatment response, a step towards metabolomics-assisted personalized healthcare. At the same time, the authors also address technical limitations such as variability in sample collection, datasets’ heterogeneity and data interpretation, and heterogeneity of the disease itself.

The study by Brown et al. explores the innovative use of tear fluid metabolomics to monitor zinc status in women participating in the BiZiFED nutritional intervention in Pakistan. Recognizing the limitations of traditional plasma zinc measurements, the researchers assessed tear metabolites collected through Schirmer strips, using LC-MS-based targeted metabolomics to quantify changes following zinc biofortified wheat consumption. The study identified significant alterations in metabolites linked to lipid metabolism, amino acid pathways, and energy metabolism. Notably, two metabolites—tiglylcarnitine and valine—showed inverse correlations with plasma zinc levels. These findings support the feasibility of using tear fluid as a non-invasive biofluid for nutritional biomarker discovery, offering new possibilities for monitoring dietary interventions and advancing global health strategies targeting micronutrient deficiencies.

The studies in this Research Topic reflect several emerging trends in ophthalmic metabolomics research. The increasing use of biofluids, such as aqueous and vitreous humor, plasma, serum, and cell culture, for metabolomic profiling represents a major advancement. The use of non-invasive fluids like tears reduces patient discomfort while enabling repeated sampling for disease monitoring. Furthermore, integrating metabolomics with other omics disciplines, such as genomics and proteomics, opens new avenues for understanding complex ocular disorders at a systems level.

Despite these promising advancements, several challenges remain. Standardization of sample collection, data processing, and metabolite identification remains a significant hurdle in ophthalmic metabolomics. Additionally, the inherent variability in metabolite concentrations due to external factors, such as diet and environmental exposures, necessitates rigorous study designs and validation cohorts. Addressing these challenges will be crucial for translating metabolomic discoveries into clinical applications.

Further advancements should include the application of artificial intelligence and machine learning in metabolomics to enhance data analysis, biomarker discovery, and facilitate predictive modelling for ocular diseases. Additionally, expanding metabolomic studies to diverse populations and animal models will help elucidate the role of metabolic pathways in ocular health and disease across different biological systems. In the case of untargeted metabolomics studies, multiplatform approaches may allow the detection of a comprehensive spectrum of metabolites, facilitating the indication of novel metabolites and disturbed metabolic pathways.

In conclusion, this Research Topic underscores the growing significance of metabolomics in ophthalmology. The studies presented herein contribute valuable knowledge and highlight the need for continued interdisciplinary collaboration to overcome existing challenges. We hope this Research Topic paves the way for further investigations to enhance our understanding of eye disorders and facilitate personalized medicine approaches for their management.

We thank all authors, reviewers, and contributors who have made this Research Topic a success. Their efforts have enriched our understanding of the role of small molecules in ophthalmic diseases and set the stage for future breakthroughs in the field.

Diana Anna Dmuchowska and Michal Ciborowski, Research Topic Editors, and Muhaiyo Bartolo Khodjayeva, Research Topic coordinator, Metabolomics in Human and Animal Ophthalmic Research.

